# 673 Pause for Progress: Implementing Antibiotic Time-Outs in Burn Care

**DOI:** 10.1093/jbcr/iraf019.302

**Published:** 2025-04-01

**Authors:** Kelly Williamson, Deepak Ozhathil, Kim Priban, Mary Bruce, Stephanie Steiner, Paula Cottrill, Anjay Khandelwal

**Affiliations:** Akron Children’s Hospital Burn Center; Akron Children’s Hospital; Akron Children’s Hospital; Akron Children’s Hospital; Akron Children’s Hospital; Akron Children’s Hospital; Adult and Pediatric Burn Institute at Akron Children’s

## Abstract

**Introduction:**

Antibiotic stewardship in burn care is critical, however to the authors knowledge, there are no standardized practices. We sought to develop a multidisciplinary antibiotic time-out (MATO) to promote shared accountability through real-time evaluation and team-based decision-making.

**Methods:**

The MATO consisted of a clinical pharmacist, burn director, nursing leadership, advanced practice provider leadership and information services. We created an electronic note that consolidated the following key metrics: indications for initiating antibiotics, culture/ laboratory testing needed, infection diagnosis, specific antibiotics to start and duration of therapy. The inclusion criteria were adult and pediatric patients admitted from April-August 2024 in whom antibiotics were started. There was a team-based review of antibiotic indications at the initiation of treatment, followed by tracking the patients’ clinical progress, culture results, and diagnoses every 72-96 hours until therapy was completed/stopped. Antibiotic orders were reviewed in the context of infection confirmation, either by culture or clinical symptoms.

**Results:**

During the study period, there were 92 patient admissions, of which 16 (average TBSA of 38.67%) met criteria, resulting in 38 MATOs, and 20 antibiotic courses evaluated. A quarter (25%) of these antibiotic courses were started due to suspicion of infection with the remaining due to confirmed infection/clinical evidence. The most common indications were wound infections with bacteremia (n=6), non-catheter related UTI (n=5), and burn cellulitis (n=4). The most common antibiotics were cefepime (n=8), vancomycin (n=4), clindamycin (n=4). The mean antibiotic duration by indication was wound infections with bacteremia (gram negative pathogen 9.8 days, gram positive pathogen 14 days), burn cellulitis 6.5 days, UTI 6.8 days. Interventions were made in 71% (n=27) of MATOs with the most common being therapeutic drug monitoring (n=10), duration (n=10), dosing (n=7), and de-escalation (n=4). All antibiotic courses were associated with a definitive indication for use.

**Conclusions:**

Integrating MATOs in a burn unit enhanced antibiotic stewardship, facilitated characterization of infections, and promoted transparent usage. The correlation between empiric treatment and confirmed indication for treatment was high; likely indicating a pre-existing culture of collaboration between providers and pharmacists. Further investigation is needed to determine how to optimize antibiotic stewardship practices across burn centers. Future interinstitutional collaboration could assist in optimizing the MATO process, definitions, and electronic note development. In addition, a comparative analysis of outcomes prior to MATO implementation would potentially validate the results.

**Applicability of Research to Practice:**

MATO programs may enhance antimicrobial stewardship across burn centers.

**Funding for the Study:**

N/A

